# Establishing Clinical Utility for Diagnostic Tests Using a Randomized Controlled, Virtual Patient Trial Design

**DOI:** 10.3390/diagnostics9030067

**Published:** 2019-06-29

**Authors:** John Peabody, Mary Tran, David Paculdo, Czarlota Valdenor, Trever Burgon, Elaine Jeter

**Affiliations:** 1 Institute for Global Health Sciences, University of California, San Francisco, CA 94158, USA; 2 Fielding School of Public Health, University of California, Los Angeles, CA 90095, USA; 3 QURE Healthcare, San Francisco, CA 94133, USA; 4 Jeter Lab Consulting, Columbia, SC 29203, USA

**Keywords:** clinical utility, diagnostics, coverage, reimbursement, randomized controlled trials, virtual patients

## Abstract

Demonstrating clinical utility for diagnostic tests and securing coverage and reimbursement requires high quality and, ideally, randomized controlled trial (RCT) data. Traditional RCTs are often too costly, slow, and cumbersome for diagnostic firms. Alternative data options are needed. We evaluated four RCTs using virtual patients to demonstrate clinical utility. Each study used a similar pre-post intervention, two round design to facilitate comparison. Representative samples of physicians were recruited and randomized into control and intervention arms. All physicians were asked to care for their virtual patients during two assessment rounds, separated by a multi-week time interval. Between rounds, intervention physicians reviewed educational materials on the diagnostic test. All physician responses were scored against evidence-based care criteria. RCTs using virtual patients can demonstrate clinical utility for a variety of diagnostic test types, including: (1) an advanced multi-biomarker blood test, (2) a chromosomal microarray, (3) a proteomic assay analysis, and (4) a multiplex immunofluorescence imaging platform. In two studies, utility was demonstrated for all targeted patient populations, while in the other two studies, utility was only demonstrated for a select sub-segment of the intended patient population. Of these four tests, two received positive coverage decisions from Palmetto, one utilized the study results to support commercial payer adjudications, and the fourth company went out of business. RCTs using virtual patients are a cost-effective approach to demonstrate the presence or absence of clinical utility.

## 1. Introduction

Advances in molecular technology and the discovery of new biomarkers are driving the development of diagnostic tests [1]. The expectation is that better diagnostics will lead to earlier, more accurate, and more specific disease treatment [2]. This promise has propelled a $7.71 billion global market projected to reach $11.54 billion by 2023 [3]. Clinicians are behind this growth, ordering an average of 16 tests but as many as 86 per Medicare patient annually [4]. All of this comes at a cost, with growing concerns from payers about what to cover and anxiety from producers on how to secure reimbursement. Despite this tension, there is fundamental agreement that only diagnostics that are clinically valid and clinically effective should have insurance coverage and reimbursement [5].

More precise guidance has recently emerged of required evidence to secure coverage and reimbursement for diagnostics. In 2011, Palmetto GBA, LLC, developed the MolDx Program and set the expectation that molecular diagnostics should not only demonstrate analytical and clinical validity, but also clinical utility for coverage approval. This program qualifies the types of data needed to demonstrate clinical utility—defined in this study as a change in a physician’s clinical practice that improves patient care and outcomes—and emphasized prospective collection of data with proper intervention and control groups [6]. Since then, other Medicare administrative contractors (MACs) and commercial insurers have adopted MolDx’s evidence standards for coverage [7]. 

For most diagnostic companies, particularly new and small ones with limited funding, proving utility to secure coverage after the arduous and expensive task of establishing validity now stands as the most significant hurdle to achieving commercial success [8]. Given these changes, the question that both payers and industry alike have to answer is: how do we collectively ensure new products are responsibly and quickly available to the right patients without stifling scientific innovation or blocking market access to life-saving technologies?

Randomized controlled trials (RCTs), in the context of demonstrated clinical efficacy, remain the gold standard for demonstrating clinical effectiveness, including clinical utility [6]. RCTs demonstrating utility, however, can be prohibitively expensive, requiring multiple investigators; a legion of patients; multiple study sites; costly infrastructure for research design, patient care, record keeping, ethical review, and statistical analysis; and several years to complete [9]. A single Phase 3 RCT can cost millions [10]. For diagnostic companies who typically lack extensive funding and are more accustomed to faster commercialization timelines, the traditional, long, and multisite equipoise RCT is out of reach [8]. Additionally, traditional patient-level RCTs are not as valuable for all study areas [11]: we believe this is the case when investigating behavioral change of the physician whose decisions are proximal to ordering the test and essential to acting upon the test results. For diagnostic utility, as a necessary first condition before a test can lead to a different intervention for the patient, an effective way to frame utility is for researchers to first demonstrate whether a new product changes provider behavior.

Several new approaches are emerging to circumvent the constraints of traditional RCTs for diagnostic companies [12]. In this paper, we review the practicality and effectiveness of conducting controlled trials for diagnostic tests, which randomize practicing physicians to care for virtual patients. These controlled trials ascertain current care standards compared to the evidence-base, adoption of the new test, and the subsequent changes in patient care recommendations. Herein, we reviewed the results and outcomes of four RCTs that used clinical performance and value (CPV) virtual patients to determine clinical utility. We used CPVs because they have been validated in peer-reviewed literature as accurate measures of actual clinical practice across any disease area [13,14]. The diagnostic tests studied are diverse: a multi-biomarker disease activity blood test for rheumatoid arthritis (RA), a chromosomal microarray assay (CMA) for developmental delays, a multiplex immunofluorescence imaging platform for prostate cancer progression, and a proteomic analysis for colorectal cancer (CRC) (Table 1). Each study used a similar experimental and easily replicable design adequately powered to assess whether the new diagnostic test demonstrated clinical utility.

## 2. Materials and Methods 

### 2.1. Study Design 

We analyzed four CPV RCTs measuring their ability to effectively demonstrate utility of a diagnostic test and report on whether they were able to successfully secure insurance coverage and reimbursement. The studies were for: (1) Crescendo Biosciences, VectraDA, (2) Lineagen, FirstStepDX PLUS CMA, (3) Metamark, ProMark and (4) Applied Proteomics, SimpliPro Colon (Table 1). The trial design, used in each of the four studies, consisted of a pre-post intervention difference-in-difference determination between a control arm and one or two intervention arm(s) with two rounds of data collection. All studies were adequately powered to detect a clinically meaningful change in behavior, defined as a 3–5% change in CPV scores [15]. Each study had a representative sample of physicians determined for each diagnostic test. Eligible providers were all formally consented before randomization. In each round, physicians cared for three randomly assigned CPV patients sent to them through an interactive online platform. Each study was conducted with the prior approval of an Institutional Review Board (IRB) and all participants were required to give written consent (Study 1: Essex IRB, #007-CO-01, Approval date: 20 April 2012; Study 2: Chesapeake IRB, #01-LIN-14, Approval date: 20 June 2014; Study 3: Chesapeake IRB, #01-MM-2014, Approval date: 11 August 2014; Study 4: Chesapeake IRB, #01-API-2016, Approval date: 21 September 2016).

For the recruitment of physicians and eligibility, in all studies, we used nationally representative lists of physicians to randomly select and serially recruit participants. Potential participants were contacted, screened, and, if eligible, invited to participate in the study until the necessary sample size for each study was met. Physicians in all studies needed to: (1) be board-certified, (2) speak English, (3) practice in a community/non-academic setting, (4) have access to the Internet, and (5) have no experience with the new test. In studies examining specialist care, specialists were required to see a minimum number of specialty patients to be eligible. Qualified and willing physicians were then randomized into control and intervention groups.

### 2.2. CPV^®^ Vignettes 

We measured clinical utility using CPV virtual patients. CPVs are well established, validated assessments of clinical practice that are responsive to any changes in practice after the introduction of a new test [15]. Each virtual patient was developed by physicians to evaluate an intended use. In these four studies we developed three different case types, each with three versions for a total of nine patients (Table S1). The case types thus evaluated the different clinical populations to determine the best use of the new diagnostic. The specific scoring criteria were evidence-based. Scoring criteria were explicit and each case had between 40 and 66 scoring criteria across all domains. Scoring of the CPVs was based on each participant’s level of adherence to these evidence-based criteria and reported as the percentage of the total scores. In addition, scores were broken down by domains of care: the history, physical exam, workup, and diagnosis plus treatment (DxTx). In each study, the new test was introduced in the workup domain. The hypothesis was that utility was demonstrated if the new test aided clinicians in reaching the right diagnosis and correct treatment plan.

### 2.3. Interventions 

Between Round 1 and Round 2, physicians randomly assigned to intervention were presented with educational materials introducing the new test. These materials came in the form of webinars and printed materials. 

In the two-arm trial design, we assigned physicians to either the intervention or control arm in an “intention-to-treat” analysis, where intervention physicians could order and be given results from the new test during Round 2. This method is preferred for determining the utility of an intervention or product. However, with CPVs, issues of messaging can also be isolated and evaluated in a unique way by adding a second intervention arm to the trial, making a three-arm trial. In the second and fourth study, the additional intervention arm received education on the new test and then gave the results of the test. Like the first intervention arm, the second intervention arm both received education on the new test. However, unlike the original intervention, the results of the new diagnostic in Round 2 were always provided for their patients. By adding a second intervention arm, the control group could be compared independently against the intervention group that received the test to determine marginal clinical utility while simultaneously being compared against the other optional intervention group to highlight and demonstrate the potential problems associated with the messaging of the product. 

### 2.4. Analysis 

Each study looked at two main outcomes: changes in overall CPV score and changes in the DxTx domain. The DxTx domain occurs after the intervention, which is provided during workup and, thus, would be the most likely to change if the diagnostic test changed clinical practice.

In all studies, we compared the outcomes in Round 2 vs. Round 1 using a difference-in-difference design with multivariate linear regression. Regression coefficients included variables for gender, age ≤40, >50% Medicare/Medicaid payer mix, high volume of specialty cases seen per week, and physician-owned practice. To determine if there was an effect from the new test, we introduced variables for the intervention group and round. The interaction term between intervention and round was the variable of interest in these models. All analyses were carried out using STATA 14.2 (Available online: https://www.stata.com/). 

## 3. Results

### 3.1. Provider Characteristics 

In total, 602 physicians participated across all four studies and each completed two CPV rounds of data collection. The baseline physician characteristics for each study is shown in Table 2. Each study pursued a provider sample that was demographically representative of the provider group nationally. Overall, greater than 95% of these community-based physicians worked four to five days per week and a vast majority worked in a physician practice group or hospital setting. Clinician types ranged from 100% primary care (study 4) to 100% specialist (studies 1, 2, and 3), based entirely on the diagnostic being studied and the targeted physician population. No significant differences between the intervention and control groups in any of the studies were found (details omitted). 

### 3.2. CPV Clinical Utility Results 

#### 3.2.1. Study 1 (VectraDA) 

In this study, we examined the utility of a multi-biomarker blood test to determine disease activity in rheumatoid arthritis (RA) among board-certified rheumatologists. Clinicians assigned to intervention were given biomarker activity scores when caring for their simulated patients. VectraDA results ranged from 0 to 100, with higher scores indicating higher disease activity and a greater need to initiate or modify non-biologic or biologic treatment for the patient. Providers were asked to care for a total of six patients across three different case types: (1) patients inadequately controlled on their current regimen who require an increased dose of their current non-biologic or the addition of another non-biologic, (2) patients inadequately controlled on their current regimen who require the addition of a non-biologic or a biologic disease-modifying anti-rheumatic drug, and (3) patients adequately treated on their current regimen for RA but with worsening symptoms due to one of four co-morbidities (depression, fibromyalgia, adenocarcinoma metastatic to bone, and osteoarthritis).

At baseline, we found no statistically significant difference in the diagnostic disease activity and treatment score between control and intervention groups (*p* = 0.09). After the introduction of VectraDA into the intervention arm, we collected a second round of cases and performed a multivariate linear regression model controlling for a number of provider and practice variables. Those providers in the intervention arm, regardless of case type, scored 12.5% higher than their control counterparts, a difference that is highly statistically significant (*p* < 0.001) (Table 3). Disaggregating by case type, we found that intervention scored higher than control for each case type (*p* < 0.05 for all), indicating use of the diagnostic provided benefits across these commonly seen case types. Of particular interest is the third case type, where a comorbidity was masquerading as disease progression, and VectraDA showed lower disease activity. By relying on this test, the intervention clinicians correctly assessed the disease severity and provided the correct treatment 14.2% (*p* < 0.001) more often than compared to controls at baseline. This meant that the intervention group was significantly less likely to incorrectly change the patient’s treatment to more expensive biologic medications (*p* = 0.008).

The study and two resulting manuscripts were completed in five months. The manuscripts were sent for peer review shortly thereafter and ultimately published in the *Journal of Clinical Rheumatology* and *PLOS One* [16,17]. Crescendo Biosciences went on to achieve a positive coverage determination by Palmetto’s MolDx program. As part of their submission, only the two clinical utility manuscripts from their CPV RCT were included in their dossier, which was enough for the initial review. Other MACs and select commercial insurance carriers followed Palmetto’s decision to cover VectraDA for their RA patients. Since their initial review, Crescendo completed a handful of studies supporting VectraDA utility, but these have come under payer scrutiny for being retrospective.

#### 3.2.2. Study 2 (FirstStep PLUS) 

In this study, pediatricians were asked to diagnose and appropriately treat pediatric patients presenting with rare genetic diseases associated with cognitive disorders. These included cases of Hunter syndrome, Mosaic Turner syndrome, SCN1 A-related seizure disorder, guanidinoacetate methyltransferase deficiency, and FOXG1 disorder. Providers in the intervention group were given results of the FirstStep Plus CMA, which described the underlying genetic abnormality and suggested treatment options. To demonstrate utility of this advanced CMA, we introduced three different case types with various levels of detectability: (1) patients where any CMA test would detect the genetic abnormality, (2) patients where only a high-resolution CMA or FirstStep would detect the abnormality, and (3) patients where only FirstStep would detect it.

At baseline, providers were only able to receive results from either a standard 180k or high-resolution CMA if they ordered it. The average overall quality of care given to their patients was 45.5%, and providers ordered CMA testing 55.7% of the time. After the intervention, when FirstStep could be ordered by intervention providers, those who did improved significantly in diagnosis and treatment (+10.9%; *p* < 0.001) compared to providers who only ordered the 180k, regular or high-resolution CMA (+2.7%; *p* = 0.122). The greatest improvement was seen in case type 2 where those who ordered FirstStep scored 10.9% better than in case type 1 (*p* < 0.001) (Table 3). 

The study and two resulting manuscripts were completed in six months. The manuscripts were sent for peer review shortly thereafter and ultimately published in *Global Pediatric Health* and *PLOS One* [18,19]. Our clinical utility manuscripts are used regularly in case approvals by commercial payers, which is their primary payer target, but not in a formal technical assessment with MolDX since this test is primarily for pediatricians. Additionally, FirstStep is now a first-tier recommendation in American College of Medical Genetics and American Academy of Pediatrics guidelines for initial evaluation of those with autism spectrum disorder, developmental delay/intellectual disability, or multiple congenital abnormalities. 

#### 3.2.3. Study 3 (ProMark) 

This study examined the clinical utility of ProMark to determine tumor aggressiveness from prostate biopsy samples guiding treatment for newly diagnosed Gleason 3 + 3 or 3 + 4 patients, an area where there is currently a lot of clinical uncertainty. We introduced three case types to determine where the proteomic assay would have its greatest utility: (A) where standard evidence (age, prostate-specific antigen (PSA), Gleason score, etc.) indicated either active surveillance (AS) or an active surgical or radiation treatment (AT) strategy that is confirmed by the assay; (B) where standard evidence indicated AS or AT but the assay recommended the opposite (i.e., switching); and (C) where standard evidence was ambiguous, and the analysis resolved the ambiguity. The primary question in this study was whether use of ProMark increased the likelihood of correct AS or AT.

At baseline across all three case types, 19.7% of physicians ordered the optimal (AS or AT) treatment and 26.0% ordered the suboptimal treatment, with the remainder leaving the choice to the patient or not recommending either option. In the second round of the study, we found that intervention providers ordered ProMark 66% of the time. Despite this, a multivariate logistic regression failed to find a significant improvement in treatment strategies for those who ordered the test (Odds Ratio (OR) = 1.06, 95% C.I. 0.55–2.03; *p* = 0.862). 

However, when we restricted our data to those who provided a definitive AS or AT course (as opposed to providing no prostate cancer treatment or solely leaving the choice to the patient), intervention providers prescribed the correct strategy 6.9% more than control providers in a difference-in-difference estimation, which was significant (*p* = 0.001), and simultaneously prescribed the suboptimal strategy 10.8% less than controls (*p* = 0.028). Overall, intervention urologists were 2.84 times more likely to provide the correct treatment than controls (*p* = 0.004) in a select subset of cases where urologists had previously outlined a definitive treatment strategy (Table 3). 

The study and one resulting manuscript were completed in six months. The manuscript was sent for peer review shortly thereafter and ultimately published in *BMC Urology* [20]. ProMark submitted a dossier for Palmetto review which only included the CPV RCT manuscript as its sole clinical utility evidence. Metamark received a positive coverage with data development determination from Palmetto, with a mandatory certification and training registry program for physicians recommending the ProMark test for Medicare patients. Since, ProMark has received positive coverage determinations from other MACs and select commercial carriers and is included in National Comprehensive Cancer Network Clinical Care Guidelines. 

#### 3.2.4. Study 4 (SimpliPro Colon) 

The fourth study looked at SimpliPro Colon, a proteomic assay of a blood sample, that determines the likelihood of CRC for elevated risk patients. Patients at risk in the US do not go for CRC screening, resulting in over 31,000 unnecessary deaths per year [21]. Most experts have assumed the problem has been the patients’ refusal to get colon cancer screening done [22]. The SimpliPro Colon assay classified patients into three buckets: (1) indeterminate—the risk of CRC for these patients is no different from the baseline population; (2) lower—the risk for these patients, although elevated versus baseline, is intermediate; and (3) higher—the risk is elevated versus baseline. We developed three different CPV patient case types, with all patients between the age of 50 and 75. Case type A were patients who were inadequately tested/screened previously (e.g., having two negative fecal occult blood test (FOBT) samples, one negative fecal immunochemical test (FIT), and a colonoscopy done 12 years ago with benign polyps). Case type B were patients who were never screened or had a distant colonoscopy and now present with symptoms suggestive of CRC in their chief complaint (e.g., meteorism, vague abdominal discomfort, change in bowel habits, weight loss, bleeding, anemia, etc.). Case type C were patients who were never screened or had a distant colonoscopy who now present with symptoms suggestive of CRC that are only identified during the review of systems (i.e., symptoms that are not the chief complaint but are uncovered as part of the routine preventive care evaluation). The results of the proteomic assay, given to the physicians in the intervention arm, were all “higher” risk. Based on guidelines and their medical history alone, these patients should have been referred for colonoscopy. 

At baseline, providers referred their A, B, and C patients for colonoscopy 71% of the time, ranging from 61% in patients presenting with abdominal distention/meteorism to 67% for those with changes in bowel habits to 84% in patients with unexplained weight loss. The baseline study upends the dogma that CRC screening is suboptimal because patients do not want to be tested. After SimpliPro Colon was introduced in the intervention group, through a series of webinars and e-materials, those in the intervention group were allowed to order it, and 23.2% did so in the post-intervention round. Compared to the control group, where 65.7% of control providers referred their patients for colonoscopy, the intervention providers who ordered the assay referred 91.8% of their patients (*p* = 0.003). By symptom type, for patients with changes in bowel habits, intervention physicians who ordered the assay were 8.2 times more likely to refer for colonoscopy compared to controls (*p* = 0.007); for abdominal distention/meteorism, they were 7.4 times more likely (*p* = 0.008); and for unexplained weight loss, they were 28.3 times more likely (*p* < 0.001). 

The study and two resulting manuscripts were completed in six months. The manuscripts were sent for peer review and published in *Current Medical Research and Opinion and the Journal of Cancer Research* and *Clinical Oncology*, respectively [23,24]. The company, however, dissolved before they could submit these findings for coverage and reimbursement. The new assay holder, who secured the technology in a liquidation sale, is using the two publications and is awaiting a commissioned third study that will extend these findings into a real-world patient assessment. This follow-on study uses the same sample frame and providers from the first two papers and looks at the CPV colonoscopy referral rates among the patients of those who adopted SimpliPro in the CPV study.

## 4. Discussion

Patient simulations that elicit real-world clinical practice patterns from active providers offers a novel, inexpensive way to reveal whether a diagnostic test provides utility in a more cost-effective way than current practices [25]. The ability of simulations to case-mix adjust and control for patient variables and to adjust the patient sample to specific clinical indications leads to cleaner provider-response signals that are more reliable and indicative of true clinical practice change. For innovative and often cash-strapped diagnostic companies, an RCT approach using validated CPV simulated patients as a practice measurement tool is an innovative way of assessing clinical utility. 

In this analysis, CPV simulated patients from four prospective RCT designs, across four very different diagnostic testing platforms that were ordered by a variety of specialists and primary care doctors. All four investigations were conducted as scientific experiments, meaning the results and the ultimate coverage and reimbursement decisions were unknown to us, the study sponsors, and our ultimate audience, the payers. 

In two of our studies (Study 1 and Study 4), we found the diagnostic test demonstrated utility for all of the patient case types targeted albeit to a varying degree. In the other two studies (Study 2 and Study 3), utility was demonstrated in only a narrow subset of the patient cases. The inclusion of multiple case types within the CPV RCT design helps companies hedge their risks of having a negative study and simultaneously allows payers to identify the most promising patient indications and approve the test only where it is likely to yield benefit to the patient. In two of the four studies, utility was demonstrated from at least one patient population in their CPV RCT, securing a positive coverage determination from MolDx.

Lack of clinical utility data is the most common reason companies fail to receive favorable coverage and reimbursement decisions [8]. The four studies herein, including the development and submission of manuscript results, were completed in six months on average and for under half a million dollars [17]. Compared to large equipoise trials that can take three to five years to complete and millions of dollars to run, CPV RCTs offer an efficient and affordable alternative to demonstrating clinical utility [19]. The failure to pursue coverage and reimbursement in a timely manner can also portend early demise for a company, particularly for newer diagnostic companies with limited resources. One of the companies, Study 4, was dissolved before they could submit these findings for coverage and reimbursement. This outcome underlines the need for early generation of evidence. The new assay holder, however, will be using the two publications developed as they reformulate their business strategy. 

Payers rightfully task diagnostic companies with proving their tests work as expected (validity) and improve clinical decision making and patient outcomes (utility), before assuming the cost and passing these costs on to the patient in the form of premiums and co-pays. However, payers also understand the realities that most diagnostic companies face shorter life cycles, limited resources, and finite funding compared to their pharmaceutical colleagues. Onerous demands for multi-year, several-hundred-patient RCTs are simply not feasible for small or newer firms that, despite their small pockets, could have the potential to save hundreds of lives. Practical criteria and feasible evidence demand for demonstrating clinical utility, such as CPV RCTs, should be strongly considered. Without this broader view, we may see great scientific advances fail to come on the market and improve the quality and cost-effectiveness of care [9]. 

Other approaches, aside from simulations and equipoise trials, have also been used to demonstrate clinical utility as well, such as the “linked evidence” [26]. We focused our analysis here on the merit of using simulations to secure coverage and reimbursement. 

There are reasons to be cautious about these results. It is a sample of four companies. There is a selection bias in that we only take companies where we have confidence the tests have proven (or pending proof of) clinical validity and that there is a clinical need for a better technology. Thus, the virtual patients are created with the clinical validity of the product confirmed. By design, not every intervention clinician orders the test. In our three-arm study, one intervention arm may order the test, while the other intervention arm is automatically given the results of the test. In effect, in the optional ordering intervention arm, we are looking at early adopters who may be systematically different from those who did not order the test. Although multiple papers have been written that show that what providers do in CPV simulations reflect what they do in real practice, the companies did not “re-prove” this with ongoing, patient-level studies, except for the fourth study. Instead, the companies elected to submit the first phase of CPV experimental results as their only level one high quality data. Finally, although our rate for obtaining coverage and reimbursement was high in our sample, coverage and reimbursement is contingent upon many factors.

## 5. Conclusions

Simulations and modeling are increasingly being used to demonstrate clinical utility of tests and drugs [27]. The goal of this review was to examine the use of simulations to assess clinical utility for a variety of new products using CPVs in an RCT design. We found that we were able to reduce case-mix, better quantify physician variability, and focus on the problem of clinical utility at a lower cost and in a shorter period of time to obtain coverage and reimbursement for selected but not for all indications.

## Figures and Tables

**Table 1:** Study sponsors and test descriptions.

Study	Company	Test
1	Crescendo Biosciences	VectraDA: blood test to assess rheumatoid arthritis activity
2	Lineagen	FirstStep^DX^ PLUS Chromosomal Microarray: high-resolution chromosomal microarray for rare disease diagnosis
3	Metamark	ProMark: multiplex immunofluorescence imaging platform analysis for aggressiveness of prostate cancer tumors
4	Applied Proteomics	SimpliPro Colon: proteomic analysis to predict likelihood of colorectal cancer

**Table 2:** Baseline characteristics by study.

Variables	Study 1	Study 2	Study 3	Study 4
	**Overall *n* = 81**	**Overall *n* = 202**	**Overall *n* = 129**	**Overall *n* = 190**
Gender (% Female)	28.4%	59.0%	2.2%	65.8%
Mean age (SD)	49.4 (9.9)	46.2 (22.8)	49.8 (8.8)	50.4 (n/a) *
Post-residency and fellowship (% years)				
0–1	2.5%	9.1%	1.4%	0.0%
2–5	16.1%	24.7%	6.5%	4.2%
6–10	19.8%	17.7%	16.6%	16.3%
11–20	19.8%	34.4%	45.3%	33.2%
21+	42.0%	14.0%	30.2%	46.3%
Practice size (% of physicians associated with practice)				
1–3	46.9%	24.2%	33.8%	44.7%
4–10	25.9%	36.1%	36.0%	34.7%
10+	27.2%	39.7%	30.2%	20.5%
Physician type (%)				
Generalist	0.0%	40.0%	0.0%	100%
Specialist	100.0%	60.0%	100.0%	0%
Single Specialty Practice (%)	59.3%	57.3%	85.5%	72.1%
Practice type (%)				
Group/Staff	n/a	66.2%	85.5%	93.2%
IPA/Network	n/a	6.3%	7.3%	6.3%
Mixed/other	n/a	27.6%	7.3%	0.5%
Practice Ownership (%)				
Physician–Physician group	71.6%	25.0%	89.2%	n/a
Hospital–Academic Medical Center	17.3%	60.9%	6.5%	n/a
Community Health Center	3.7%	7.3%	3.6%	n/a
Other	7.4%	6.8%	0.7%	n/a
Employed by practice (% Yes)	77.8%	93.3%	66.2%	73.7%
Average days worked per week (%)				
1–3	2.5%	10.9%	0.0%	n/a
4	33.3%	22.9%	10.9%	n/a
5+	64.2%	66.2%	89.1%	n/a
Proportion of all patients covered by				
Medicare	39.2%	7.3%	47.4%	32.1%
Commercial	46.6%	44.6%	41.2%	52.4%
Medicaid	7.7%	40.6%	6.4%	7.6%
Self-pay	4.9%	5.2%	3.7%	5.8%
Other	1.6%	2.8%	1.3%	2.1%

SD: Standard Deviation; n/a: information is not available; * estimated average age, based on age groups.

**Table 3:** Clinical utility demonstration of each novel diagnostic.

Outcome	Coefficient	*p*-Value
Linear Regression *		
VectraDA—Intervention DxTx Score Improvement over Baseline	12.5%	<0.001
FirstStep—Intervention DxTx Score Improvement over Baseline	10.9%	<0.001
Logistic Regression *		
ProMark—OR Intervention provides correct AS or AT to patient	2.84	0.004
SimpliPro Colon—OR Intervention orders diagnostic colonoscopy for patient	3.88	<0.001

* Multivariate model accounting for provider and patient characteristics; OR: Odds Ratio; AS: Active Surveillance; AT: Active Treatment

## Supplementary Materials

The following are available online at https://www.mdpi.com/2075-4418/9/3/67/s1, Table S1: Clinical performance and value (CPV) patient cases for the four studies.
