# Supplementary material for: Establishing Clinical Utility for Diagnostic Tests Using a Randomized Controlled, Virtual Patient Trial Design
Source: Diagnostics (Basel). 2019 Jun 29;9(3):67. doi: 10.3390/diagnostics9030067 (PMC6787613; doi:10.3390/diagnostics9030067)
Supplement: Supplementary file 1 [file diagnostics-09-00067-s001.pdf]

Table S1. CPV Patient Cases for the Four Studies.

Study 1 (VectraDA) –.

Case Number	Summary Description
1	46/F, seen for follow up of her rheumatoid arthritis diagnosed 4 months ago. Currently, reports persistent morning stiffness and worsening pain in the balls of her feed and toes. Further evaluation reveals mild-moderate rheumatoid arthritis (RA) requiring addition of methotrexate.
2	45/F, seen for follow up of her RA diagnosed 8 weeks ago. Currently, reports morning stiffness lasting an hour and some difficulty at work with pain and fatigue when working the morning shift. Workup reveals moderate RA requiring addition of another DMARD.
3	35/F, second follow up visit for RA diagnosed 5 months ago. Reports consistent morning stiffness lasting 30 to 45 minutes. Workup reveals moderate RA requiring addition of methotrexate.
4	52/M, seen for follow up after initial diagnosis of RA 4 months ago. Has pain in most MCP and PIP joints, feet, and ankles with morning stiffness for about 45 minutes. Workup demonstrates progressive, moderate RA requiring addition of a biologic or synthetic DMARD.
5	55/M, diagnosed with RA 8 months ago. Currently with distinct tenderness and redness in the small joints of his hands and wrists with 1-2 hours of stiffness in the morning. Workup reveals a progressive, moderate-to-severe RA requiring switching to another biologic or adding another DMARD.
6	50/F, diagnosed with RA 6 months ago. Currently, she has return of pain over both hands and wrists, accompanied by 45 minutes of morning stiffness. Evaluation reveals progressive, moderate RA requiring adding a biologic or synthetic DMARD.
7	72/F, diagnosed with RA 4 months ago. Her symptoms have waxed and waned but have not flared to the same extent since she has been on DMARD therapy. The results of her evaluation reveal a waning, mild RA requiring no changes to her RA treatment.
8	65/F, diagnosed with RA 9 months ago. Currently complaining of worsening pain, weakness, and no energy, with pain in her wrists, hands feet, elbows, shoulders, hips, and knees. Workup reveals waning, mild RA requiring no change to her RA treatment.
9	66/M, diagnosed with RA 2 years ago and is 5 months status post prostatectomy for adenocarcinoma. Complaining of mild-to-moderate left shoulder pain increasing in intensity in the last 2 months. Working reveals waning, mild RA requiring no change to her RA treatment.

Study 2 (FirstStepDx) –.

Case Number	Summary Description
1	5/M with limited motor, social, and language skills along with recurrent ear and respiratory infections. Ultimately, diagnosed with Hunter syndrome.
2	12 months/F presenting with myoclonic seizures and failure to thrive. Diagnosed with guanidinoacetate methyltransferase deficiency.
3	18 months/F with delayed language and gross motor milestones and facial anomalies. Diagnosed with Mosaic Turner syndrome with XY cell line.
4	8 months/F with delayed motor and social milestones with hands in consistent plantarflexion. Diagnosed with congenital Rett (FOXG1) syndrome.
5	18 months/M with myoclonic seizures and developmental decline. Diagnosed with SCN1A/Dravet syndrome.
6	5 months/M with generalized spasms and hypotonia. Diagnosed with X-linked lissencephaly with abnormal genitalia
7	5/M with limited motor, social, and language skills along with recurrent ear and respiratory infections. Diagnosed with Bloom syndrome
8	12 months/F presenting with myoclonic seizures and failure to thrive. Confirmed high risk for ASDs because of RAB11-FIP5 gene mutation and deletion at 1q41.
9	18 months/F with delayed language and gross motor milestones and facial anomalies. Diagnosed with VHL disease type 2B.

Study 3 (ProMark) –.

Case Number	Summary Description
1	60/M, reporting lower urinary tract symptoms for three years. His diabetes is controlled on medications. PSA results stable in the past 3 years. A recent biopsy showed Gleason 6 adenocarcinoma with 2 of 12 biopsy cores positive, less than 50% cancer in both cores. Further workup showed a more indolent course and ProMark recommendation of active surveillance.
2	81/M, reports single episode of gross hematuria and recurrent lower back pain for the past 2 weeks. PSA results showed increasing trend (2.9 one year ago, 5.5 at 6 months, and 9.8 currently). Biopsy showed Gleason 6 adenocarcinoma with 5 of 12 cores positive and less than 50% cancer in all cores. Workup showed a nonaggressive course and ProMark recommendation of active surveillance.
3	73/M, seen for hesitancy and frequency of urination that has worsened over the past 6 months. 2 months ago, he was noted to have a PSA of 13.8. Biopsy revealed Gleason 3+4 with 20% of the tumor having Gleason 4 morphology. ProMark recommends active surveillance.
4	57/M, coming in for his ED. Biopsy revealed Gleason 3+4 with 45% of tumor having Gleason 4. ProMark results recommend the possibility of active surveillance.
5	55/M, reports symptoms of prostatism. Biopsy revealed Gleason 6 with 4 of 12 cores positive for adenocarcinoma. ProMark result supports active surveillance.
6	63/M, seen post discharge from hospital to be evaluated for a suspicious DRE, linked to a PSA of 8.9. Biopsy showed 6 of 12 cores positive for Gleason 6 adenocarcinoma with 70% of tumor positive for cancer. ProMark results supports switch to active treatment.
7	62/M, seen for gross hematuria after initial negative US except for enlarge prostate.Biopsy revealed T2a lesion with 3 of 12 cores positive, Gleason 3+4, with only 10% showing Gleason 4. ProMark recommends switch to active surveillance.
8	81/M, seen for suspicious nodularity on his prostate. PSA was stable for 6 months. Biopsy revealed 6 of 12 cores positive for cancer with 70% tumor positive, Gleason 7. ProMark recommendation of switch to active treatment
9	77/M, seen for regular check up. Initial PSA was 22.1 and biopsy showed 1 of 12 cores was positive for adenocarcinoma, Gleason 3+4, with 50% have Gleason 4. ProMark recommends switching to active treatment.

Study 4 (SimpliPro Colon) –.

Case Number	Summary Description
1	67/F comes in for annual checkup. Reports feeling well except for intermittent constipation over the past 6 months. Had a screening colonoscopy when she was 55 y/o, with findings of 2 hyperplastic polyps removed from the sigmoid colon
2	53/M comes in on the one-year anniversary of his quitting smoking. For the past 4 weeks, he notes having recurrent episodes of abdominal discomfort and gas that lasts 15-30 minutes before resolving on their own. Has never had a screening colonoscopy but did have one negative FIT done 3 years ago.
3	52/M is a usually healthy patient who is seen for his annual checkup. He reports losing 8 pounds over the past 3-4 months. Had a FOBT test performed two years ago with negative results
4	57/F comes to clinic for refills on her emphysema medication. Her emphysema is under good control with no exacerbations in the past 2 years. She complains of having intermittent episodes of diarrhea alternating with constipation over the past 2 months, despite not having changed her diet. She has never had a FIT, FOBT or other colorectal screening test performed.
5	64/M seeks opinion regarding episodes of abdominal distention and excess flatulence that has been bothering him for the past 2 weeks. OTC medications did not help his symptoms. He has not undergone any cancer screening.
6	68/F comes in for 2 episodes of hematochezia in the past 4 days. She denies having any other symptoms. She says her stools are blood-streaked and that some blood is admixed with brown stool. She has not had a colonoscopy or any other means of CRC Screening
7	55/F, with well-controlled DMII and diabetic kidney disease, comes in for her annual blood tests and pneumococcal/influenza shots. On review of systems, she tells you that her bowel movements have been less regular in the past 3 weeks (every other day instead of her usual daily bowel movements) and that she has been having occasional headaches relieved by resting, but is otherwise negative for other complaints. She has not undergone a colonoscopy or other form of colorectal cancer screening.
8	60/M comes in for leg edema. Review of systems is negative except for intermittent abdominal discomfort and fullness over the past 2 months or so. Bowel movements described as unremarkable. He has never been compliant to his appointments for colonoscopy given his "busy schedule" and has never been interested in providing a stool sample.
9	61/F seen for follow-up for osteoporosis and fibromyalgia. On review of systems, she mentions feeling fatigued more in the afternoon and needs to rest or even takes a short nap but didn't really feel it was something to be worried about. She also notes that she has seen some blood in her stool and that it is dark.
